# Complete Genome Sequence of the Red-Pigmented Strain Serratia marcescens SCQ1 and Its Four Spontaneous Pigment Mutants

**DOI:** 10.1128/MRA.01456-20

**Published:** 2021-04-15

**Authors:** Tingting Xiang, Rui Liu, Jing Xu, Cailing Xu, Nuo Wang, Wei Zhou, Yu Zhao, Minhui Luo, Yongji Wan

**Affiliations:** a Laboratory of Invertebrate Pathology and Applied Microbiology, College of Sericulture, Textile and Biomass Sciences, Southwest University, Chongqing, China; University of Delaware

## Abstract

Serratia marcescens SCQ1 is a red-pigmented bacterium isolated from silkworm larva with septicemia. Pigment-deficient spontaneous mutants arise when S. marcescens SCQ1 is incubated under relatively stable laboratory conditions for a long time. Here, we present the complete genome sequence of SCQ1 and the resequenced genomes of four spontaneous pigment mutants.

## ANNOUNCEMENT

Serratia marcescens, a kind of Gram-negative bacterium, belongs to the family *Enterobacteriaceae*, which is widely distributed in diverse environments, including water, soil, plants, and animals (1). Some strains of S. marcescens may produce a red pigment, prodigiosin, which is a secondary metabolite with multiple bioactivities that has a linear tripyrrole skeleton (2-methyl-3-pentyl-6-methoxyprodiginine) (2–4). S. marcescens SCQ1 was isolated from the hemolymph of diseased silkworm larvae in a sericultural area of Chongqing, China. The hemolymph of silkworm larvae was streaked onto an LB plate and incubated at 28°C for 24 h. Red colonies were picked and restreaked onto LB medium 3 times to obtain the axenic strain S. marcescens SCQ1, which was stored at −20°C. Strain SCQ1 was then passaged 20 times in liquid LB medium (pH 7), incubated at 28°C with shaking (200 rpm). Every day, 500 μl of culture was transferred into 50 ml of fresh LB. After 20 transfers, 50 μl of the diluted cultures was plated onto LB medium. Colonies with different pigment phenotypes were picked, and the mutant strains SCQ1-1M (pink), SCQ1-2M (white), SCQ1-3M (white), and SCQ1-4M (white sides and red center) were characterized by different colony colors (5). The genetic changes underlying the pigment deficiencies in the S. marcescens mutants have not yet been identified.

S. marcescens SCQ1 and the other four spontaneous mutants were cultured in LB broth at 28°C. Genomic DNA was extracted using the E.Z.N.A. bacterial DNA kit (Omega Bio-tek, Norcross, GA). DNA concentration and quality were assessed using a NanoDrop 2000 UV-visible (UV-vis) spectrophotometer (Implen, CA, USA) and 1% agarose gel electrophoresis. Genomic DNA of SCQ1 was sheared into ∼10-kb fragments using a g-TUBE device (Covaris). A library was constructed for SCQ1 using the SMRTbell Express template preparation kit v2.0 (Pacific Biosciences, USA) and sequenced using single-molecule real-time (SMRT) sequencing technology on the PacBio RS II sequencing platform (6). A total of 3,152,565,048 bases and 320,553 subreads were generated. The mean subread length and *N*_50_ value were 9,834 bp and 11,305 bp, respectively. The reads were assembled using Falcon v2.0.0. Genomic DNA from SCQ1-1M, SCQ1-2M, SCQ1-3M, and SCQ1-4M was sheared into 400- to 500-bp fragments using the M220 sonicator (Covaris, Woburn, MA, USA). Paired-end sequencing libraries were prepared for SCQ1-1M, SCQ1-2M, SCQ1-3M, and SCQ1-4M using the NEBNext Ultra DNA library prep kit (NEB, USA). Whole-genome sequencing of SCQ1-1M, SCQ1-2M, and SCQ1-3M was performed using the HiSeq X Ten platform (Illumina, San Diego, CA, USA), and whole-genome sequencing of SCQ1-4M was performed using the Illumina HiSeq 4000 platform to generate 2 × 150-bp paired-end reads. Quality control on the Illumina data was assessed with FastQC v0.11.5 (7). The adapter sequences, low-quality reads, and reads containing more than 10% of N bases were removed. Default parameters were used except where otherwise noted.

The assembled SCQ1 genome sequence was 5,063,944 bp with an average coverage of ∼622× and a GC content of 61.33%, and it consists of a 4,994,354-bp circular chromosome and a 69,590-bp plasmid. The genome was annotated using the National Center for Biotechnology Information (NCBI) Prokaryotic Genome Annotation Pipeline (PGAP) v4.13 (8). It contains 4,692 protein-encoding genes, 22 rRNA genes, 91 tRNA genes, 14 noncoding RNA (ncRNA) genes, 25 pseudogenes, and 1 CRISPR array. We predicted 7 genomic islands using IslandPath-DIOMB v0.2 software (9) and 4 prophage sequences using PhiSpy v2.3 software (10). For whole-genome sequencing of SCQ1-1M, SCQ1-2M, SCQ1-3M, and SCQ1-4M, we obtained 20,976,838, 23,788,882, 18,999,970, and 8,876,328 clean reads, respectively. Using the Burrows-Wheeler Aligner (BWA) v0.7.8 (11), the reads were aligned to the SCQ1 assembled genome sequence. We used SAMtools v0.1.18 (12) for converting the SAM and BAM formats and sorting, indexing, and calling variants. Identified variants were annotated using the ANNOVAR v2019-09-01 tool (13). The resequencing depth was at least 184× with coverage of more than 99.99%. In total, 26 single nucleotide polymorphisms (SNPs) were detected, including 8 in SCQ1-1M, 8 in SCQ1-2M, 8 in SCQ1-3M, and 2 in SCQ1-4M. All of them were substitutions of base, and no indels were detected in the genomes of the mutants. No SNPs were found in the prodigiosin biosynthetic gene cluster in strains SCQ1-2M, SCQ1-3M, and SCQ1-4M. In strain SCQ1-1M (pink), one SNP was found in the *pigJ* gene. This SNP is a nonsynonymous substitution (A to G), which caused the change of an amino acid from Asp to Gly and may impact the activity of PigJ (beta-ketoacyl synthase).

### Data availability.

The complete genome sequence of S. marcescens SCQ1 has been deposited in GenBank under accession numbers CP063354 (chromosome) and CP063355 (plasmid). The raw sequencing data for SCQ1, SCQ1-1M, SCQ1-2M, SCQ1-3M, and SCQ1-4M have been deposited in the NCBI SRA database under BioProject accession number PRJNA386063 and SRA accession numbers SRR12825186, SRR12737688, SRR12825185, SRR12825184, and SRR12825183, respectively.
